# Genome sequences of *Acinetobacter* phage At2 and its host *Acinetobacter tandoii* W4-4-4

**DOI:** 10.1128/mra.00009-25

**Published:** 2025-05-22

**Authors:** Soon Keong Wee, Biaoguo Yan, Su'Aidah Binte Mustaffa, Eric Peng Huat Yap

**Affiliations:** 1 Institute for Digital Molecular Analytics and Science, Nanyang Technological University 54761, Singapore, Singapore; 2 Lee Kong Chian School of Medicine, Nanyang Technological University 54761, Singapore, Singapore; 3 School of Biological Sciences, Nanyang Technological University 54761, Singapore, Singapore; Queens College Department of Biology, Queens, New York, USA

**Keywords:** *Acinetobacter*, bacteriophages, environmental microbiology

## Abstract

We present the genome sequences of *Acinetobacter* phage At2 and its host *Acinetobacter tandoii* strain W4-4-4, both isolated from a tropical mangrove swamp in Singapore. This report highlights the genetic features of both organisms, including antimicrobial resistance genes.

## ANNOUNCEMENT

First identified from activated sludge in Australia (1), *Acinetobacter tandoii* has also been found in termite gut microbiota and exhibits phenol degradation properties (2). Bacteriophages coexist with their host bacteria in the natural environment (3, 4), driving evolution and potentially enhancing its bioremediation applications, such as phenol degradation. We report the draft genomes of *Acinetobacter tandoii* strain W4-4-4 and lytic *Acinetobacter* phage At2 co-isolated from brackish water at an urban coastal swamp in Singapore.

The bacterial host was isolated through sequential culture in Baumann enrichment medium (5), Leeds acinetobacter medium agar (6), and lysogeny broth agar at 37°C incubation. The phage was subsequently isolated from the same sample by plaque formation on the host monoculture using double-layer agar with 0.6% soft agar. Antibiotic susceptibility was assessed by disk diffusion following CLSI standards (7). DNA from the bacterial colony and phage lysate was extracted using QIAGEN DNA Mini Kit. Concentrations were measured by Nanodrop and Qubit HS dsDNA Assay. Genomes were sequenced using Nextera XT DNA library preparation on the Illumina HiSeq platform with 150 bp paired-end reads, yielding 5,293,391 host reads and 773,186 phage reads. Quality check and adaptor trimming were performed using FastQC v0.72 and Trimmomatic v0.36.5 (8, 9). *De novo* assembly was performed with Unicycler Galaxy version 0.4.6.0 (10, 11). Host species identification was done using dDDH analysis in TYGS Server (12). Host and phage genomes were annotated using NCBI Prokaryotic Genome Annotation Pipeline 4.9 (13) and Prokka 1.13 (14), respectively. Antimicrobial resistance genes, virulence factors, CRISPR arrays, and phage regions were predicted using ResFinder 3.2 (15), VFDB VFanalyzer (16), CRISPRCasFinder (17), and PHASTEST (18). Default parameters were used for all software tools.

*Acinetobacter tandoii* strain W4-4-4 has a 3,575,445 bp genome (GC content, 40.15%). The draft assembly, in 50 contigs (coverage depth, 373×; *N*_50_, 312,393 bp), contains 3,276 coding DNA sequence (CDS) genes, 60 pseudogenes, 66 tRNAs, and 3 rRNAs. It carries the *bla*_OXA_ gene, sharing 92.63% nucleotide identity with *bla*_OXA-664_, an intrinsic carbapenemase in *Acinetobacter tandoii* (19). Phenotypically, it is susceptible to various antibiotics including carbapenems (Table 1). No CRISPR arrays were detected, but two intact prophage regions (34.5 kb to 38 CDS and 39.1 kb to 43 CDS) and one questionable prophage region were identified.

**TABLE 1:** Antibiotic susceptibility testing phenotype of *Acinetobacter tandoii* W4-4-4

Class	Phenotype^*a*^
Aminoglycoside	S: amikacin and gentamicin
Beta-lactam	S: ampicillin/sulbactam, piperacillin/tazobactam, doripenem, imipenem, meropenem, ceftazidime, and cefotaxime
Quinolone	S: ciprofloxacin
Sulfonamide	S: trimethoprim/sulfamethoxazole
Tetracycline	S: tetracycline
Trimethoprim	S: trimethoprim/sulfamethoxazole

^*a*^ S: susceptible.

**Fig 1:**
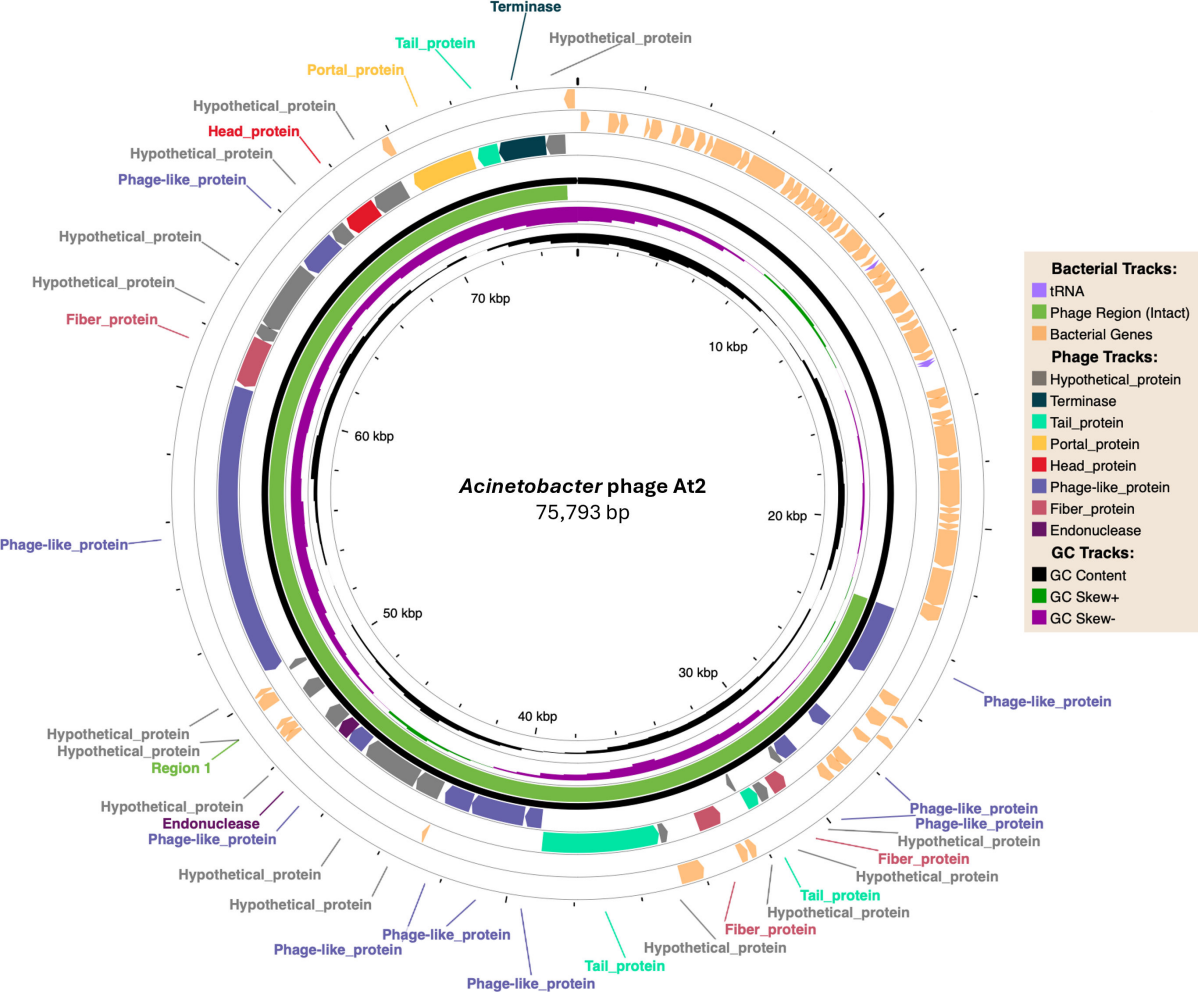
Gene annotation plot of *Acinetobacter* phage At2 generated using PHASTEST (18).

*Acinetobacter* phage At2 has a 75,793 bp circular genome (GC content, 39.47%; coverage depth, 3,131×) (Fig. 1). It encodes 99 CDS and 2 tRNAs, with 87 (87.8%) being hypothetical proteins. It features two N4-like RNA polymerases (virion-associated RNA polymerase and RNA polymerase II) and a single-stranded DNA-binding protein, suggesting a genome injection mechanism akin to phage N4 (20). Other replication-associated genes encode DNA polymerase I, DNA helicase, DNA primase, ATPase, ribonucleotide reductase, and thymidylate synthetase. Structural proteins identified include baseplate hub, tail fibers, and tail terminase components. Homology with N4 proteins suggests a conserved N4-like genome injection and replication mechanism (21). Classified within the *Caudoviricetes* class, Phage At2 shares 67% and 70% amino acid identity with two *Acinetobacter* phages from the *Schitoviridae* family infecting *Acinetobacter beijerinckii* (phage nACB1) and *Acinetobacter pittii* (phage VB_ApiP_XC38), respectively.

## Data Availability

The whole-genome sequences have been deposited in GenBank/DDBJ/ENA under accession numbers GCA_008867985 (W4-4-4 bacterial genome assembly) and PQ656108 (At2 phage sequence). The isolates have been deposited under BioSample accession numbers SAMN12769619 and SAMN46355607 under BioProject accession numbers PRJNA565663 and PRJNA1214016. The raw reads are available in the Sequence Read Archive (SRA) under accession numbers SRR32146046 and SRR32073887, respectively.
